# Solid histological component of adenocarcinoma might play an important role in PD‐L1 expression of lung adenocarcinoma

**DOI:** 10.1111/1759-7714.14209

**Published:** 2021-11-23

**Authors:** Tomoyuki Miyazawa, Kei Morikawa, Kanji Otsubo, Hiroki Sakai, Hiroyuki Kimura, Motohiro Chosokabe, Naoki Furuya, Hideki Marushima, Koji Kojima, Masamichi Mineshita, Junki Koike, Hisashi Saji

**Affiliations:** ^1^ Department of Chest Surgery St. Marianna University School of Medicine Kawasaki Japan; ^2^ Division of Respiratory Medicine, Department of Internal Medicine St. Marianna University School of Medicine Kawasaki Japan; ^3^ Department of Pathology St. Marianna University School of Medicine Kawasaki Japan

**Keywords:** early adenocarcinoma, immunohistochemistry, lung cancer, PD‐L1, subtype

## Abstract

**Background:**

In this study we aimed to clarify the PD‐L1 positive expression in lung adenocarcinoma, including various adenocarcinoma subtypes paying particular attention to its component.

**Methods:**

A total of 307 lung adenocarcinoma patients who underwent lobectomy or segmentectomy, as well as systematic lymph node dissection (ND2a), from February 2008 to March 2020 at our hospital, were enrolled into the study. A final diagnosis of adenocarcinoma was obtained from the resected lung specimens of all 307 patients to determine the histological type, adenocarcinoma subtype, and component of adenocarcinoma by ethics of 5%. PD‐L1 was immunohistochemically stained using the murine monoclonal antibody clone 22C3.

**Results:**

When PD‐L1 expression‐positive was defined by tumor proportion score (TPS) ≥1%, the positive cases were 6/33 in adenocarcinoma (Ad) in situ (AIS), 2/26 in minimally invasive Ad (MIA), 12/60 in lepidic predominant Ad (LPA), 44/91 in papillary predominant Ad (PPA), 24/49 in acinar predominant Ad (APA), 23/28 in solid predominant Ad (SPA), 4/7 in micropapillary predominant Ad (MPA), and 0/13 in invasive mucinous Ad (IMA). In the high proportion group (APA, PPA, SPA, and MPA) of PD‐L1 expression, SPA was the only subtype which was statistically significant when both PD‐L1 expression‐positive was defined by TPS ≥ 1% (*p* < 0.0001) and TPS ≧ 50% (*p* < 0.0001). We then considered the solid component. We investigated 279 cases of the other subtype group excluding SPA. The group containing a solid component (≥5%) tended to be PD‐L1 expression‐positive both when defined by TPS ≥1% (*p* < 0.0001) and TPS ≧50% (*p* = 0.0049).

**Conclusions:**

The PD‐L1 expression tended to be positive when a solid component was confirmed (≥5%) in specimens of lung adenocarcinoma patients.

## Graphical abstract

When solid component was confirmed even a little (5%≥) in the lung adenocarcinoma patients specimen, PD‐L1 expression tend to positive.

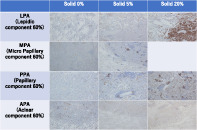

## INTRODUCTION

Lung cancer is the leading cause of cancer deaths in most developed countries worldwide.^1^ Despite multidisciplinary therapies that have been used for patients with advanced non‐small cell lung cancer (NSCLC), the overall survival rates are still unsatisfactory, especially when genetic mutation is not detected. Recently, several humanized monoclonal antibodies to block immune checkpoints have been developed which have proven to be useful in selected patients with unresectable NSCLCs.^2^, ^3^ The association between programmed cell death 1 (PD‐1) and programmed death‐ligand 1 (PD‐L1) can target these monoclonal antibodies. Inhibition of the PD‐1/PD‐L1 axis enhances antitumor immunity in order to prevent tumor cells escaping from host immune responses, thereby providing a promising strategy for effective tumor immunotherapy.^4^ Previous clinical trials have revealed treatment effect predictor of immune checkpoint inhibitors (ICIs), such as PD‐L1 expression^5^, ^6^, ^7^ and tumor mutation burden^8^ are positively correlated, and SKT11 and KEAP1 mutations are negatively correlated.^9^ Therefore limited and controversial debates still continue today. On the other hand, pembrolizumab is an ICI which can be used for single agent administration in the first treatment of patients with advanced NSCLC. The tumor proportion score (TPS) has been found to be the most correlated effect predictor of pembrolizumab.^5^, ^7^, ^10^

ICIs have been approved as consolidation therapy after radiation chemotherapy for locally advanced stage disease,^11^ and expected to be expanding the indication in the region of treatment for more early stage lung cancer,^12^, ^13^ such as radiation combination therapy,^14^ pre‐ and postoperative therapy. In particular, with regard to early stage lung cancer, in the near future, we may be able to judge adaptation of pre‐ and postoperative ICI therapy according to the pathological and genetic findings obtained from specimens at diagnosis. To date, preoperative biopsy using bronchoscopy is used to diagnose histological subtypes of lung adenocarcinoma.^15^ To clarify the adenocarcinoma subtype which tends to express PD‐L1 is important. We have previously reported on the relationship between PD‐L1 expression and adenocarcinoma subtypes using 78 resected cases.^16^ In order to obtain more precise knowledge, in this study we used a greater number of resected lung cancer specimens to investigate the histological component of adenocarcinoma.

## METHODS

### Patients and pathological specimens

A total of 307 lung adenocarcinoma patients (148 men and 159 women; age range = 42–90 years; mean age = 69.2 years) who underwent lobectomy or segmentectomy, together with systematic lymph node dissection (ND2a), from February 2008 to March 2020 at our hospital were enrolled into this study. The exclusion criteria were patients who had undergone preoperative induction therapy. The mean postoperative follow‐up period was 26.8 months (range = 0–121 months). The TNM stages of patients were determined according to the international staging criteria for lung cancer published by the International Association for the Study of Lung Cancer (IASLC) in 2017 (eighth edition). Of 34 patients with stage IB disease, 27 patients (79.4%) received postoperative adjuvant therapy with oral uracil tegafur (UFT), of 35 patients with stage II–III, 14 (40.0%) received intravenous platinum doublet‐based chemotherapy and two patients received pembrolizumab with or without platinum doublet as a clinical trial (MK3475‐671). Forty‐six patients relapsed, then 11 patients received standard anticancer therapy according to the guidelines. Nine patients received EGFR‐TKIs, and 16 patients received ICIs as treatment for recurrence. Of 46 patients, 14 patients (30.4%) did not receive treatment for recurrence, and 26 patients (56.5%) received first‐line therapy, five (10.9%) patients received second‐line therapy, and one (2.1%) patient received third‐line therapy.

A final diagnosis of adenocarcinoma was obtained from resected lung specimens of all 307 patients by two pathologists in order to determine the histological type, adenocarcinoma subtype, and component of adenocarcinoma by ethics of 5% based on the World Health Organization (WHO) pathological classification published in 2015.^17^ The pathological stages were: 0 in 19 patients, IA (IA1–IA3) in 216, IB in 33, IIA in five, IIB in 14, IIIA in 14, IIIB in two, IVA in one, and IVB in one.

Subtypes of adenocarcinoma were Ad in situ (AIS) in 33 patients, minimally invasive Ad (MIA) in 26, lepidic predominant Ad (LPA) in 60, papillary predominant Ad (PPA) in 91, acinar predominant Ad (APA) in 49, solid predominant Ad (SPA) in 28, invasive mucinous Ad (IMA) in 13 and micropapillary predominant Ad (MPA) in seven. This study was approved by the ethics committee of St. Marianna University School of Medicine, Kanagawa, Japan (accession no. 5090). All patients provided written informed consent.

### Immunohistochemistry

**FIGURE 1:**
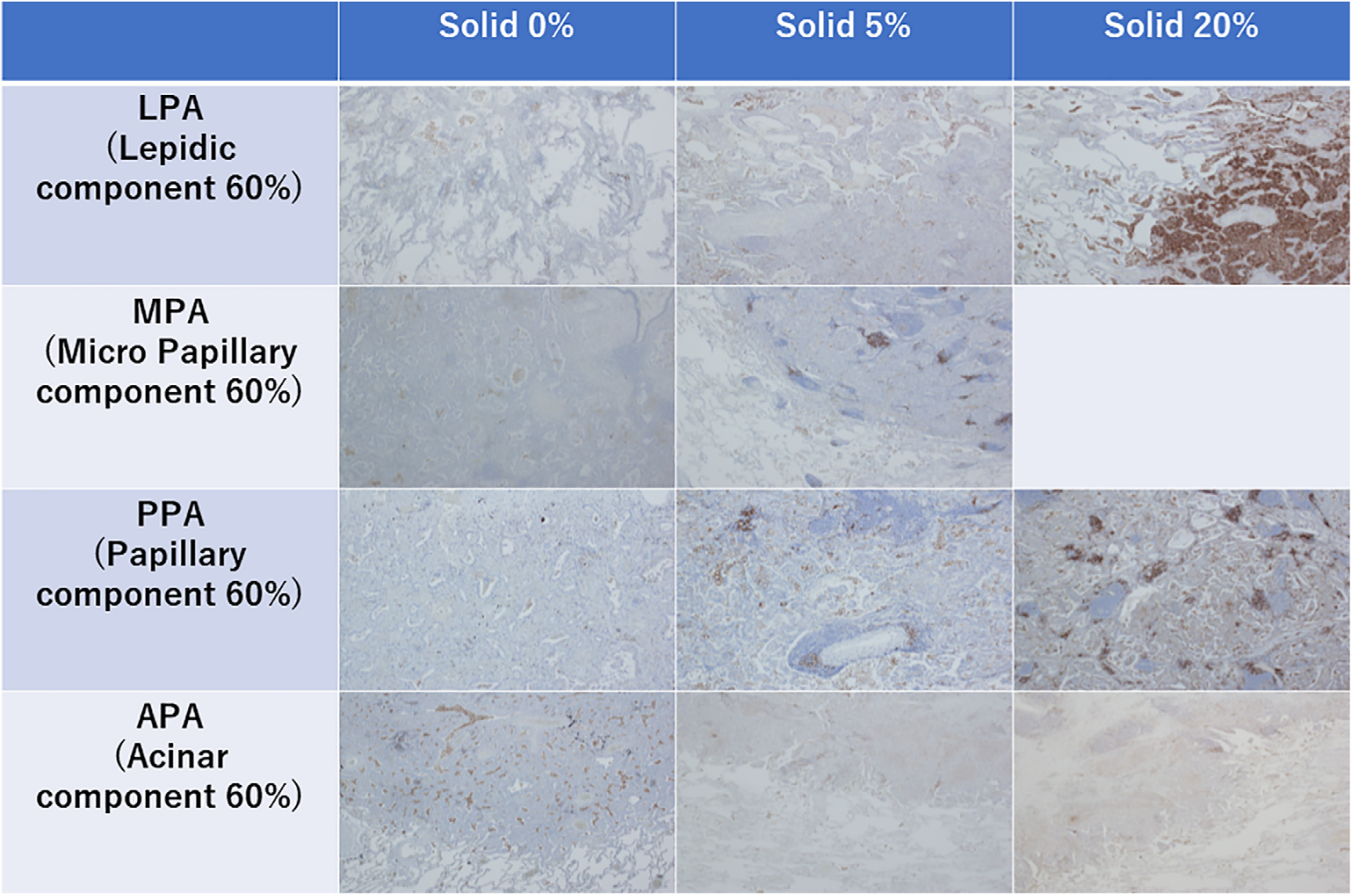
Staining of PD‐L1‐positive tumor cells of various subtypes (LPA, MPA, PPA and APA). Specimens show a predominant component 60%, and solid components 0%, 5%, and 20%. There were no cases of MPA containing a solid component 20%. APA, acinar predominant adenocarcinoma (Ad); LPA, lepidic predominant Ad; MPA, micropapillary predominant Ad; PPA, papillary predominant Ad

Immunohistochemistry was performed using the PD‐L1 kit (PD‐L1 IHC 22C3 pharmDX; Dako) according to the manufacturer's instructions. This antibody was selected since the Food and Drug Administration (FDA) approved this system as a companion diagnostic test to determine the applicability of treatment using pembrolizumab. In brief, serial 3 μm‐thick tissue sections were cut from formalin‐fixed, paraffin‐embedded blocks. Sections were deparaffinized in xylene and rehydrated through a graded series of ethanol concentrations. Antigen retrieval was carried out using a 97°C water bath for 20 min in Envitio FLEX Target Retrieval solution (Dako). Intrinsic peroxidase activity was blocked using hydrogen peroxide for 5 min. After washing the section with a wash buffer (Dako), primary antibodies were applied to cover the specimen. Sections were incubated at room temperature for 30 min. After three washes in the wash buffer for 5 min each, slides were incubated with antimouse linker antibody specific to the host species of the primary antibody, and were then incubated with a ready‐to‐use visualization reagent consisting of secondary antibody molecules and horseradish peroxidase molecules coupled to a dextran polymer backbone. Specimens were then counterstained with hematoxylin for 5 min and cover‐slipped (Figure 1).

### Assessment of PD‐L1 expression

We followed the instructions given in the PD‐L1 Immunohistochemistry Testing in Lung Cancer manual of the IASLC (https://www.iaslc.org/sites/default/files/wysiwyg-assets/pd-l1_atlas_book_lo-res.pdf). Briefly, the authors counted PD‐L1‐positive tumor cells, defined as complete circumferential or partial cell membrane staining. Cytoplasmic staining and tumor‐associated immune cells, such as macrophages, were excluded from scoring. The tumor proportion score (TPS) was calculated as follows:

TPS(%) = (PD−L1−positive tumor cells / Total number of tumor cells) × 100

The TPS was used to categorize the staining status as follows: <1% (negative staining), ≥1% and <50% (weakly positive staining), and ≥50% (highly positive staining).

### Statistical analysis

Statistical analysis was performed using JMP software. Clinicopathological characteristics by categorical variables were evaluated using the chi‐square test. *p* < 0.05 was considered statistically significant. The overall survival (OS) and recurrence‐free survival (RFS) were analyzed using the Kaplan–Meier method.

## RESULTS

**FIGURE 2:**
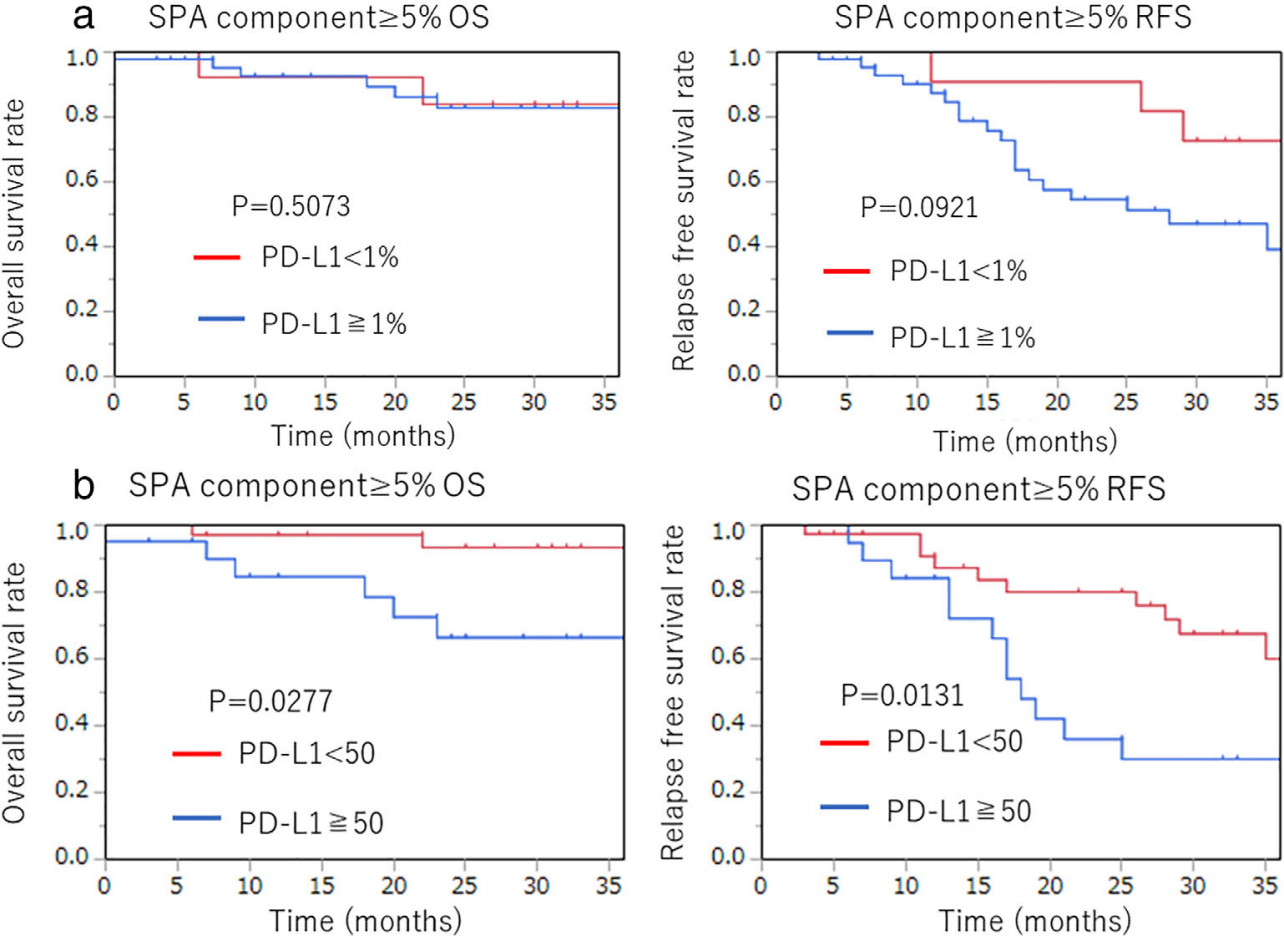
Kaplan–Meier survival curves. (a) There was no statistical significance between TPS ≧ 1% or not, both in OS (*p* = 0.5073) and RFS (*p* = 0.0921) in adenocarcinoma including solid component. (b) There was a statistical significance between TPS ≧ 50% or not, both in OS (*p* = 0.0277) and RFS (*p* = 0.0131) in adenocarcinoma including a solid component. OS, overall survival; RFS, recurrence‐free survival; TPS, total protein score

Clinicopathological characteristics and the percentages of PD‐L1 expression (TPS < 1%, 50%>TPS ≥ 1%, and TPS ≧ 50%) patients are shown in Table 1. PD‐L1 positivity was higher in males (*p* < 0.0001), smokers (*p* < 0.0001), advanced pathological stages ≥IB (*p* = 0.0049), pathological nodal status (pN1‐3 or pN0) (*p* = 0.0269), positive venous invasion (*p* < 0.0001), positive lymphatic invasion (*p* = 0.0011), and positive pleural invasion (p1‐3 or p0) (*p* = 0.0112). However, age (≥75 or <75), *EGFR* mutation status, carcinoembryonic antigen (CEA) (≧5 ng/ml or not), and sialyl Lewis X‐i antigen (SLX) (≧38 U/ml or not) were not associated with PD‐L1 expression. Limited to Ad subtypes, when TPS≧1% was PD‐L1 positive, PPA (48.4%), APA (49.0%), SPA (82.2%), and MPA (57.1%) were higher than in AIS (18.2%), MIA (7.7%), LPA (26.7%), and IMA (0%). Therefore, we independently compared PD‐L1 expression of PPA, APA, SPA, and MPA with compared to others (shown in Table 2). APA and MPA were not significantly different either when PD‐L1 positive was defined as TPS ≧ 1% and TPS ≧ 50%. PPA was significantly different only if PD‐L1 positive was defined as TPS ≧ 1% (*p* = 0.0261), not in TPS ≧ 50% (*p* = 0.7384). Only SPA was significantly different both in TPS ≧ 1% (*p* < 0.0001), and in TPS ≧ 50% (*p* < 0.0001). Therefore, we hypothesized that the solid component plays an important role in PD‐L1 expression. In this study, we investigated the component of adenocarcinoma by ethics of 5%, whereby 5% was the minimum percentage component judged included. In all cases (including SPA), when comparing the solid component group (solid component ≥ 5%) with the other groups, we found a significant difference both in TPS ≧ 1% (*p* < 0.0001), and in TPS ≧ 50% (*p* < 0.0001). Apart from SPA, comparing the solid component group (solid component ≥ 5%) with the other groups, we were able to confirm that there was a significant difference both in TPS ≧ 1% (*p* < 0.0001), and in TPS ≧ 50% (*p* = 0.0049) (Table 3). Regarding the prognosis of patients with a solid component (SPA + solid component ≥5%), Kaplan–Meier survival curves revealed that there was a statistically significant difference both in the OS and RFS by a high PD‐L1 expression (OS; *p* = 0.0277, RFS; *p* = 0.0131). However, this was not the case in patients with a weak PD‐L1 expression (OS; *p* = 0.5073, RFS; *p* = 0.0921) (Figure 2). Multivariate analysis showed the presence of a solid component and smoking status were independent predictive factors for PD‐L1 expression (Table 4).

**TABLE 1:** Clinicopathological characteristics and PD‐L1 expression (*n* = 307) in adenocarcinoma lung cancer patients

	Total 307	TPS < 1 (%)	1% ≦ TPS < 50 (%)	TPS ≧ 50 (%)	*p*‐value
Gender					
Male	148	70	56	22	<0.0001^*^
Female	159	118	32	9	
Age					
≧75	106	59	31	16	0.0979
<75	201	129	57	15	
Smoking					
Current/former	162	75	61	26	<0.0001^*^
Never	145	113	27	5	
Histological subtype					
AIS	33	27	6	0	
MIA	26	24	2	0	
LPA	60	44	12	4	
IMA	13	13	0	0	
PPA	91	47	35	9	
APA	49	25	21	3	
SPA	28	5	8	15	
MPA	7	3	4	0	
Pathological stage					
p0‐IA3	236	156	61	19	0.0049^*^
IB‐	71	32	27	12	
Pathological nodal status					
pN0	286	181	78	27	0.0269^*^
pN1‐	21	7	10	4	
Venous invasion					
v0	265	177	68	20	<0.0001^*^
v1‐	42	11	20	11	
Lymphatic invasion					
ly0	275	178	73	24	0.0011^*^
ly1‐	32	10	15	7	
Pleural invasion					
pl0	267	171	74	22	0.0112^*^
pl1‐	40	17	14	9	
*EGFR* mutation					
Positive	65	42	18	5	0.8884
Negative	92	57	26	9	
Not examined	150				
CEA					
CEA≧5.0	17	10	5	2	0.9764
<5.0	285	173	83	29	
Not examined	5				
SLX					
SLX ≧ 38	32	16	12	4	0.4873
<38	249	152	71	26	
Not examined	26				

Abbreviations: AIS, adenocarcinoma in situ; APA, acinar‐predominant invasive adenocarcinoma; CEA, carcinoembryonic antigen; EGFR, epidermal growth factor receptor; IMA, invasive mucinous adenocarcinoma; LPA, lepidic‐predominant invasive adenocarcinoma; MIA, minimally invasive adenocarcinoma; MPA, micropapillary predominant invasive adenocarcinoma; PPA, papillary‐predominant invasive adenocarcinoma; SLX, sialyl Lewis X‐i antigen; SPA, solid‐predominant invasive adenocarcinoma; TPS, tumor proportion score.

^*^ Statistically significant.

**TABLE 2:** Comparison of PD‐L1 expression of APA, PPA, SPA and MPA

	*n*	TPS ≧ 1%	*p*‐value	TPS ≧ 50%	*p*‐value
APA	49	24 (49.0%)	0.1128	2 (4.1%)	0.0928
Non‐APA	258	95 (36.8%)		29 (11.2%)	
PPA	91	44 (48.4%)	0.0261^*^	10 (11.0%)	0.7384
Non‐PPA	216	75 (34.7%)		21 (9.7%)	
SPA	28	23 (82.1%)	<0.0001^*^	15 (53.5%)	<0.0001^*^
Non‐SPA	279	96 (34.4%)		16 (5.7%)	
MPA	7	4 (57.1%)	0.3205	0	0.2194
Non‐MPA	300	115 (38.3%)		31 (10.3%)	

Abbreviations: APA, acinar‐predominant invasive adenocarcinoma; MPA, micropapillary predominant invasive adenocarcinoma; PPA, papillary‐predominant invasive adenocarcinoma; SPA, solid‐predominant invasive adenocarcinoma; TPS, tumor proportion score.

^*^ Statistically significant.

**TABLE 3:** Comparison of PD‐L1 expression of adenocarcinoma containing a solid component

	*n*	TPS ≧ 1%	*p*‐value	TPS ≧ 50%	*p*‐value
Containing solid component (SPA + solid ≥ 5%)	60	46 (76.7%)	<0.0001^*^	21 (35.0%)	<0.0001^*^
Not containing solid component (solid < 5%)	247	73 (29.6%)		10 (4.1%)	
Adenocarcinoma without SPA					
Containing solid component (solid ≥ 5%)	32	23 (71.9%)	<0.0001^*^	6 (18.8%)	0.0049^*^
Not containing solid component (solid < 5%)	247	73 (29.6%)		10 (4.1%)	

Abbreviations: SPA, solid‐predominant invasive adenocarcinoma; TPS, tumor proportion score.

^*^ Statistically significant.

**TABLE 4:** Multivariate analysis using Cox regression model for recurrence‐free survival

	*p*
Gender	0.1272
Smoking	0.0463*
Containing solid component (SPA + solid ≥ 5%)	<0.0001*
Pathological stage	0.8115
Pathologic nodal status	0.9584
Venous invasion	0.0722
Lymphatic invasion	0.7622
Pleural invasion	0.5263

Abbreviation: SPA, solid‐predominant invasive adenocarcinoma.

^*^ Statistically significant.

## DISCUSSION

Lung cancer management is becoming more individualized for age, comorbidities, cancer type, stage, and patient preference.^18^ Immunotherapy is one of the most expected fields of individualized management for NSCLC. Many study results regarding PD‐L1 protein expression in NSCLC have been reported. However, there are very few studies which refer to adenocarcinoma subtypes. To the best of our knowledge, this is the first study concerning the relationship between PD‐L1 expression and adenocarcinoma component. With regard to immunotherapy, preoperative neoadjuvant immunotherapy may become one of the options for NSCLC management in the future.^19^, ^20^ The technical progression of bronchoscopic biopsy may support this trend.^21^ Today, adenocarcinoma subtypes can be diagnosed by examining the specimen obtained by bronchoscopic biopsy. Matsuzawa et al., who analyzed the concordance rate of histological subtypes between small biopsy samples and surgically‐resected specimens, stated concordance rate of PPA and SPA were relatively high.^15^ Therefore, solid compartment of biopsy‐based analysis may also have more reliability than the other components. This fact gives more importance to this report. Regarding the frequency of PD‐L1 expression in adenocarcinoma subtypes, when cutoff TPS was ≧ 1%, the highest positive rate of PD‐L1 expression was observed in the SPA (73.7%), followed by the APA (49.2%), PPA (43.3%), and MPA (40.0%). This result is almost concordant with 404 cases reported by Song et al.^22^ Peng reported when cutoff TPS was ≧ 25%, the highest positive rate of PD‐L1 expression was observed in the SPA (44.7%), followed by APA (25.1%), MPA (20%), and PPA (10.9%). When cutoff TPS was ≧ 50%, the highest positive rate of PD‐L1 expression was also observed in the SPA (53.6%) in this study. In any case, SPA tend to contribute positive PD‐L1 expression. With regard to subtypes, we previously reported that adenocarcinoma subtypes are divided into three groups by prognosis^23^ (poor, MPA + SPA; moderate, APA + PPA + IMA; good, AIS + MIA + LPA). When cutoff TPS was ≧ 1%, the poor group was 27/35 (77.1%), the moderate group was 68/153 (44.4%), and the good group was 24/119 (20.2%). When cutoff TPS was ≧ 50%, the poor group was 15/35 (42.9%), the moderate group was 12/153 (7.8%), and the good group was 4/119 (3.4%). Positive rate was concordant with poor prognosis. Despite IMA belonging to the moderate group, the positive rate of IMA was 0%. It might be because of a shortage of cases, because just 13 IMA cases were included in this study. With regard to prognosis, Kaplan–Meier survival curves showed that there was a statistically significant difference both in OS and RFS by high PD‐L1 expression in patients with only a little solid component (SPA + solid component ≥5%), but not by those with weak PD‐L1 expression. Because PD‐L1 expression tends to be concordant with a worse prognosis (smoking history, advanced pathological stage, venous invasion, lymphatic invasion, pleural invasion, and worse subtype group), higher PD‐L1 expression may be related to a worse prognosis. However, in the near future, it is likely that high PD‐L1 expression in patients with a solid component might have a better prognosis following ICI treatment, in the same way that patients with *EGFR* mutation might have a better prognosis following EGFR‐TKI treatment.^24^

In the era of individualized therapy for lung cancer, when specimens including those with a solid component can be obtained by bronchoscopic biopsy before surgery, preoperative neoadjuvant immunotherapy might become one of the options in the treatment of adenocarcinoma. Further, in inoperable cases, when specimens including a solid component can be obtained, PD‐L1 expression may be positive.

In conclusion, there was only a significant difference in PD‐L1 expression in SPA both in TPS ≧ 1%, and in TPS ≧ 50%. Even when a solid component was confirmed (≥5%) in the lung adenocarcinoma patient specimens, PD‐L1 expression tended to be positive. Therefore, a solid histological component of adenocarcinoma might play an important role in PD‐L1 expression of lung adenocarcinoma. There are several limitations in this study. First, subtype diagnosis in adenocarcinoma is not always coincident between pathologists.^25^, ^26^ Second, this was not a prospective study, and therefore bias might exist. Third, the postoperative observation period was relatively short and the number of cases relatively small. Further studies including larger numbers of patients are necessary to confirm the present results. Fourth, this study includes old specimens, and the accuracy of PD‐L1 staining results might therefore be insufficient.

## CONFLICT OF INTEREST

The authors do not report any conflict of interest.
